# Implications for Dementia Policy Advocacy and Design: Lessons from Walking the Talk for Dementia 2024

**DOI:** 10.1002/alz70860_097739

**Published:** 2025-12-23

**Authors:** Walter D Dawson, Fernando Aguzzoli Peres, Jonathan Adrian Zegarra‐Valdivia, Lingani Mbakile‐Mahlanza, Kate Irving, Sherril B Gelmon

**Affiliations:** ^1^ Global Brain Health Institute, San Francisco, CA, USA; ^2^ Oregon Health & Science University, Portland, OR, USA; ^3^ Portland State University, Portland, OR, USA; ^4^ Global Brain Health Institute, Dublin, Dublin, Ireland; ^5^ Universidad Señor de Sipán, Chiclayo, Peru; ^6^ University of Botswana, Gaborone, Botswana; ^7^ Dublin City University, Dublin, Ireland

## Abstract

**Background:**

Global efforts to advance dementia policy and advocacy are increasing. Dementia policy change is particularly salient to low‐ and middle‐income countries, which are experiencing the world's largest growth in dementia prevalence. Walking the Talk for Dementia (WTD) is an opportunity to advance more equitable dementia policy on a global scale. WTD convenes key parties across the dementia continuum including researchers, clinicians, people living with dementia, care partners, policy‐influencers, artists, and advocates who can collectively influence global and national dementia policies.

**Method:**

Policy and advocacy‐related data were collected from the WTD 2024 participants from 27 countries. Data sources included 95 written/audio/video reflections from 54 participants, gathered during the event and a post‐event survey (*N* = 73; 87% response rate). Reflections and survey responses were coded by pairs of research team members. Thematic analysis was utilized to identify opportunities for the WTD experience to influence dementia advocacy and policy.

**Result:**

Participation in the WTD facilitated several opportunities to influence dementia advocacy and the design of policy. These include extended time to create relationships leading to new collaborations between professionals and people with lived experience of dementia; greater awareness of the multiple dimensions of the lived experience of dementia reducing dementia‐related stigma; and future strategies to ensure the meaningful engagement of people living with dementia and care partners within advocacy and policy efforts.

**Conclusion:**

The experiences, insights gleaned, and connections made among WTD participants have the potential to influence global dementia policy and advocacy. The primary pathways for influence are through an increased awareness of the experiences of people living with dementia and their care partners, reductions in dementia stigma, and cross‐national collaborations among new partners. Efforts to influence the design and development of dementia‐related policy must include people living with dementia and care partners. This approach can influence national plans for dementia and specific policy areas such as the financing of care and support, funding of dementia research, explicit recognition and support of care partners, and public health awareness campaigns to address stigma and dementia risk reduction, reflecting and valuing the voices of people with lived experience of dementia.

